# Neuronal Profilin Isoforms Are Addressed by Different Signalling Pathways

**DOI:** 10.1371/journal.pone.0034167

**Published:** 2012-03-28

**Authors:** Kai Murk, Nina Wittenmayer, Kristin Michaelsen-Preusse, Thomas Dresbach, Cora-Ann Schoenenberger, Martin Korte, Brigitte M. Jockusch, Martin Rothkegel

**Affiliations:** 1 Cellular Neurobiology, Zoological Institute, TU Braunschweig, Braunschweig, Germany; 2 Department of Anatomy and Cell Biology, Center of Anatomy, Georg August University Göttingen, Göttingen, Germany; 3 M.E. Müller Institute for Structural Biology, University of Basel, Basel, Switzerland; 4 Cell Biology, Zoological Institute, TU Braunschweig, Braunschweig, Germany; University of Edinburgh, United Kingdom

## Abstract

Profilins are prominent regulators of actin dynamics. While most mammalian cells express only one profilin, two isoforms, PFN1 and PFN2a are present in the CNS. To challenge the hypothesis that the expression of two profilin isoforms is linked to the complex shape of neurons and to the activity-dependent structural plasticity, we analysed how PFN1 and PFN2a respond to changes of neuronal activity. Simultaneous labelling of rodent embryonic neurons with isoform-specific monoclonal antibodies revealed both isoforms in the same synapse. Immunoelectron microscopy on brain sections demonstrated both profilins in synapses of the mature rodent cortex, hippocampus and cerebellum. Both isoforms were significantly more abundant in postsynaptic than in presynaptic structures. Immunofluorescence showed PFN2a associated with gephyrin clusters of the postsynaptic active zone in inhibitory synapses of embryonic neurons. When cultures were stimulated in order to change their activity level, active synapses that were identified by the uptake of synaptotagmin antibodies, displayed significantly higher amounts of both isoforms than non-stimulated controls. Specific inhibition of NMDA receptors by the antagonist APV in cultured rat hippocampal neurons resulted in a decrease of PFN2a but left PFN1 unaffected. Stimulation by the brain derived neurotrophic factor (BDNF), on the other hand, led to a significant increase in both synaptic PFN1 and PFN2a. Analogous results were obtained for neuronal nuclei: both isoforms were localized in the same nucleus, and their levels rose significantly in response to KCl stimulation, whereas BDNF caused here a higher increase in PFN1 than in PFN2a. Our results strongly support the notion of an isoform specific role for profilins as regulators of actin dynamics in different signalling pathways, in excitatory as well as in inhibitory synapses. Furthermore, they suggest a functional role for both profilins in neuronal nuclei.

## Introduction

The actin cytoskeleton determines birth, maintenance, function and structural plasticity of neuronal synapses. In the presynapse, an actin filament meshwork regulates the release and recycling of neurotransmitter containing vesicles [1]. At the postsynapse, actin is involved in converting neuronal activity into structural changes (reviewed in [2]). Thus, the morphology of dendritic spines, the postsynaptic structures that mainly receive the excitatory input, depends on the dynamics of actin [3] that in turn is regulated by numerous actin-binding proteins.

Prominent regulators of neuronal actin dynamics are profilins (reviewed in [4]). In the mammalian and avian CNS, two isoforms, profilin 1 (PFN1) and profilin 2a (PFN2a), are co-expressed [5], [6], with PFN2a contributing up to 75% of the total profilin [7]. PFN1 is expressed in all mammalian cells, but in quite variable amounts in different brain regions [8]. In addition to a general role in neuritogenesis [9], [10], it may exert specific functions in neuronal subpopulations [10]. Biochemical data demonstrated interactions of PFN1 and PFN2a with pre- and postsynaptic proteins [11], [12], [13], [14], [15].

Genetic, physiological and biochemical studies have led to controversal interpretations on the role of PFN2a in synaptic architecture and function. Biochemical data revealed PFN2a associated with effectors of exocytotic and endocytotic pathways [6] and suggested its involvement in the assembly of the endocytotic machinery [16]. Furthermore, a mouse mutant with a deleted *pfn2* gene displays an increase in synaptic vesicle exocytosis [8], consistent with an inhibitory role for PFN2a in the control of presynaptic membrane trafficking. On the other hand, overexpressed PFN2a was observed to translocate into dendritic spines of cultured neurons in an activity-dependent manner [17], [18], and fear conditioning correlated with profilin enrichment in dendritic spines of rat amygdalae [19]. Hence, both studies suggested an important, if not unique role for PFN2a at the postsynapse.

More recent findings showed that PFN1 and PFN2a have overlapping as well as differential effects on dendritic architecture: The physiological level of PFN2a is essential for normal dendritic complexity and spine numbers, but in neurons with decreased PFN2a, PFN1 can only rescue spine numbers, not dendritic complexity [20].

To unravel the functional differences between PFN1 and PFN2a in more detail, we first determined their endogenous levels in synaptic structures of cultured rodent neurons, in sections of mature rat cortex, hippocampus and cerebellum and in neuronal nuclei. Using isoform specific monoclonal antibodies in immunofluorescence and immunoelectron microscopy, we detected both isoforms in the same neuronal compartment. Furthermore, we report that they differentially respond to changes in neuronal activity. These data reveal that PFN1 and PFN2a are linked to different signalling pathways.

## Results

### Primary hippocampal neurons express both PFN isoforms in the same synaptic structures

**Figure 1:**
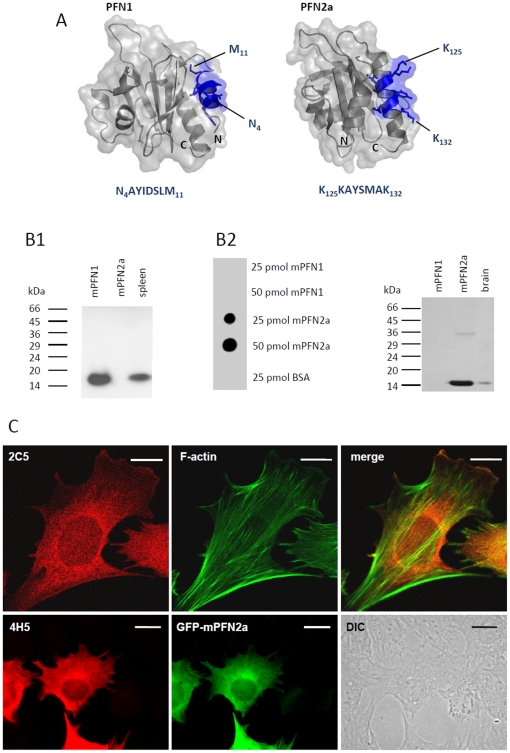
Specificity of the monoclonal antibodies raised against PFN1 (2C5) or PFN2a (4H5). (A): Isoform-specific epitopes recognised by the monoclonal antibodies 2C5 and 4H5, as determined by pepscan analysis on overlapping 15mer amino acid sequences, as described [51]. Their respective location on the surface of PFN1 or PFN2a (blue) is indicated in the structural models designed by using Pymol software (DeLano Scientific LLC, Palo Alto, USA, version 0.98). (B): PFN isoform specific reactivity of 2C5 and 4H5. (B1): Immunoblot of 2C5 with purified recombinant mouse PFN1, mouse brain PFN2a and total extract of mouse spleen. (B2): Dot blot (left) and immunoblot (right) of 4H5 with recombinant mouse PFN1, mouse brain PFN2a and total brain extract. BSA: bovine serum albumin used as control. (C): Confocal images of immunofluorescence with 2C5 (upper panels) and 4H5 (lower panels) of C2C12 mouse myoblasts transfected with GFP-PFN2a. The antibody 2C5 reveals a typical fine diffuse cytoplasmic, but also a nuclear staining for PFN1, while the PFN2a specific antibody 4H5 labels only the GFP-PFN2a transfected cells (lower left) as part of a C2C12 cell population (DIC image; lower right). Filamentous actin was stained with FITC-phalloidin. Bars: 10 µm.

**Figure 2:**
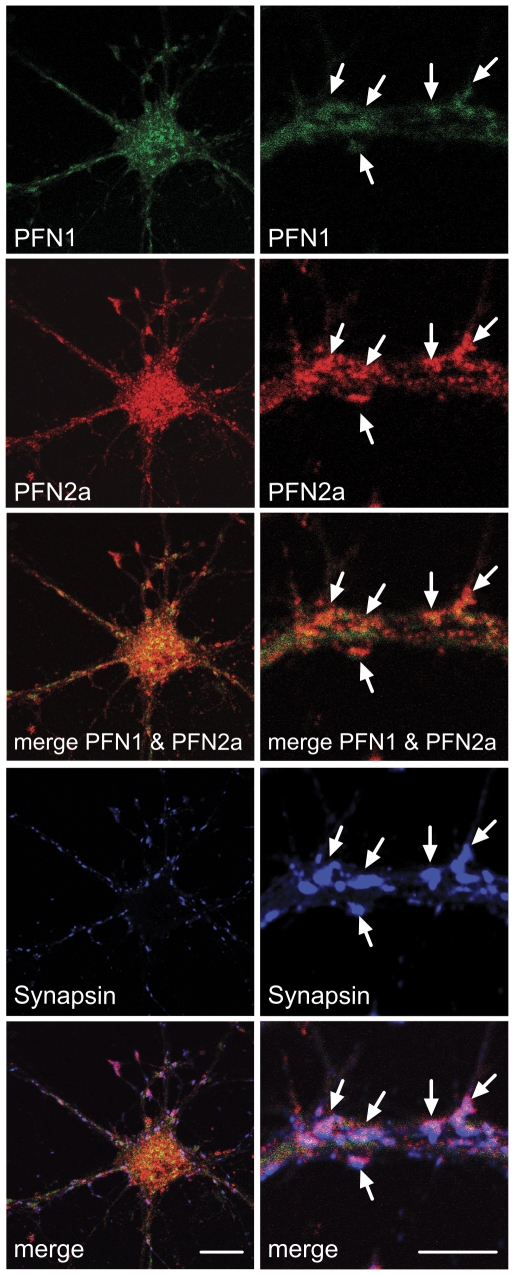
Cell bodies and synaptic structures of cultured embryonic neurons contain profilin 1 (PFN1) and profilin 2a (PFN2a). Confocal images showing the simultaneous immunostaining of PFN1 and PFN2a in a mouse hippocampal neuron (DIV14) with monoclonal antibodies specific for each isoform, and with a rabbit serum specific for the synaptic markers synapsin 1 and 2. Note that both isoforms are concentrated in synapses (arrows, right panels). Bar: 10 µm.

To visualise both profilin isoforms in cultured embryonic neurons, we used a pair of monoclonal antibodies (mABs) specific for PFN1 and PFN2a, (Figure 1). The antibody mAB 4H5, generated against bovine brain PFN2a, recognises a PFN2a-specific epitope at the C-terminus in mammals and in birds [5], while mAB 2C5 is directed against an N-terminal peptide sequence of PFN1 that was fused to a nanoparticle carrier [21] By pre-labelling of one mAB and using two different fluorophores (see Material and Methods) we localised PFN1 and PFN2a simultaneously in the same cultured embryonic neuron (DIV14) derived from murine hippocampus (Figure 2), and counterstaining for synapsin revealed both isoforms within the same synaptic structures. In general, the PFN1 signal was also seen along the neurites, while PFN2a was more strictly confined to synapses (Figure 2).

### Both profilin isoforms are predominantly localised in the postsynaptic compartment of different brain regions

**Figure 3:**
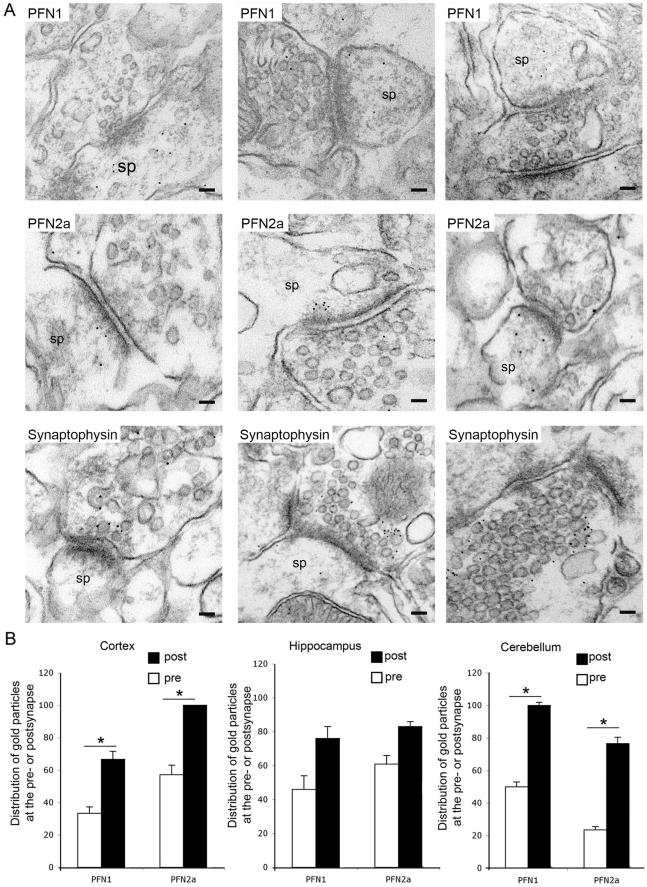
PFN1 and PFN2a are enriched at postsynaptic sites in the adult rat brain. (A): Ultrastructural localisation of PFN1 (upper row) and 2a (centre row) in the cortex (left column), CA1 region of the hippocampus (centre column) and cerebellar cortex (right column) as seen with pre-embedding immunogold labelling. Immunogold labelling of synaptophysin (bottom rows), a presynaptic marker, served to demonstrate the localisation of both profilin isoforms in synapses. Sp: dendritic spine. Bars: 50 nm. (B): Quantitation of PFN1 and PFN2a immunoreactivity in pre- and postsynaptic structures in cortex, hippocampus and cerebellum. The Y-axis represents the percentage of pre- and postsynapses positive for gold particles. Note that both isoforms are more densely concentrated in postsynaptic than in presynaptic structures. (25–50 synapses per experiment, mean errors are based on 2 independent experiments; * P<0.05, statistical analysis by paired *t-*test).

For an unequivocal assignment of PFN1 and PFN2a to pre- and postsynaptic structures, we performed immunoelectron microscopy on ultrathin sections derived from different regions of the adult rat brain. The presynaptic compartment was identified by the density of neurosecretory vesicles, and corroborated by pre-embedding immunogold labelling with antibodies against synaptophysin (Figure 3A) whereas adjacent structures were recognised as postsynaptic spines. Immunogold labelling with anti-PFN1 and anti-PFN2a revealed both proteins in both compartments (Figure 3A). Notably, quantitation of the gold particles showed that both isoforms were more abundant in post- than in presynapses. Moreover, PFN2a labelling was more prominent than PFN1, with a noticeable accumulation at the cytoplasmic face of the postsynaptic density, similar to the previously reported localisation of the profilin-ligand Mena [22] (Figure 3B). These data corroborate previous reports demonstrating that PFN2a is more frequent in rodent brain than PFN1 [7].

### PFN2a colocalises with gephyrin clusters at inhibitory synapses

**Figure 4:**
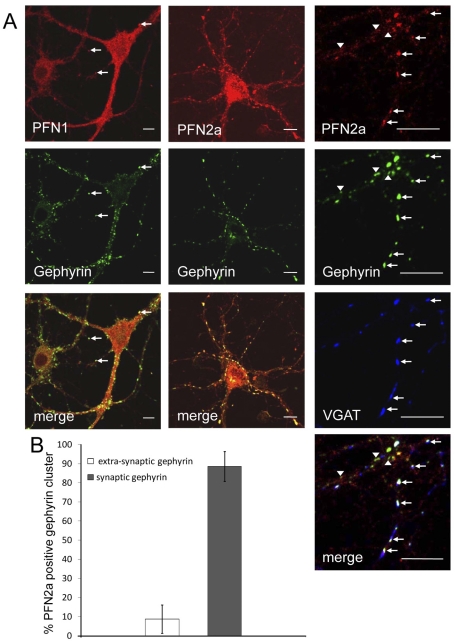
PFN2a is enriched in postsynaptic regions of inhibitory synapses. (A): Confocal images of neurons with simultaneous immunostaining of PFN1 (left column), PFN2a (centre column) and gephyrin, a protein concentrated in the active zone of inhibitory postsynapses. (cf. [2]). Note that PFN2a is frequently concentrated in gephyrin clusters, while PFN1 is rarely enriched in these structures (arrows). Right column: Higher magnification of a neuron immunostained for PFN2a, gephyrin and VGAT, a marker for the inhibitory presynapse. Note that PFN2a primarily colocalises with synaptic gephyrin clusters (arrows), whereas extra-synaptic gephyrin clusters, identified by lack of VGAT staining, are mostly negative for PFN2a (arrow heads). Bar: 10 µm. (B): Quantitative analysis of the presence of PFN2a in synaptic and extra-synaptic gephyrin clusters (at least 483 extra-synaptic and 1830 synaptic gephyrin clusters per experiment, mean errors are based on 3 independent experiments, statistical analysis by unpaired *t* test).

The higher abundance of PFN2a in postsynaptic than presynaptic structures of different brain regions led us to study profilins in different subtypes of synapses. To reveal the isoform localisation in inhibitory synapses, we again labelled cultured mouse hippocampal neurons with the isoform-specific mABs simultaneously, and counterstained the samples with an antibody against gephyrin, a component of the postsynaptic protein network of inhibitory synapses and a ligand of both profilin isoforms [12]. PFN1 and PFN2a were both detected in inhibitory synapses (Figure 4), but they clearly differed in their extent of co-localisation with gephyrin: PFN1 was diffusely spread throughout dendrites and only occasionally accumulated at gephyrin clusters, whereas PFN2a was primarily concentrated in bright spots that coincided with gephyrin clusters along dendrites (Figure 4A, left and centre columns). Because gephyrin is not confined to the postsynaptic compartment [23], [24], we counterstained PFN2a- and gephyrin-labelled neurons for the presynaptic marker VGAT that is restricted to inhibitory synapses [25]. We found PFN2a preferentially associated with large gephyrin clusters adjacent to VGAT-positive synaptic terminals, whereas smaller extra-synaptic gephyrin spots were mostly negative for PFN2a (Figure 4, right column). Quantitation of the PFN2a, VGAT and gephyrin signals confirmed that in inhibitory neurons, PFN2a is primarily concentrated at the synaptic gephyrin clusters of postsynaptic terminals. Figure 4B shows that gephyrin clusters not associated with synapses comprise only approximately 10% of the total.

### The levels of profilins correspond to synaptic activity

**Figure 5:**
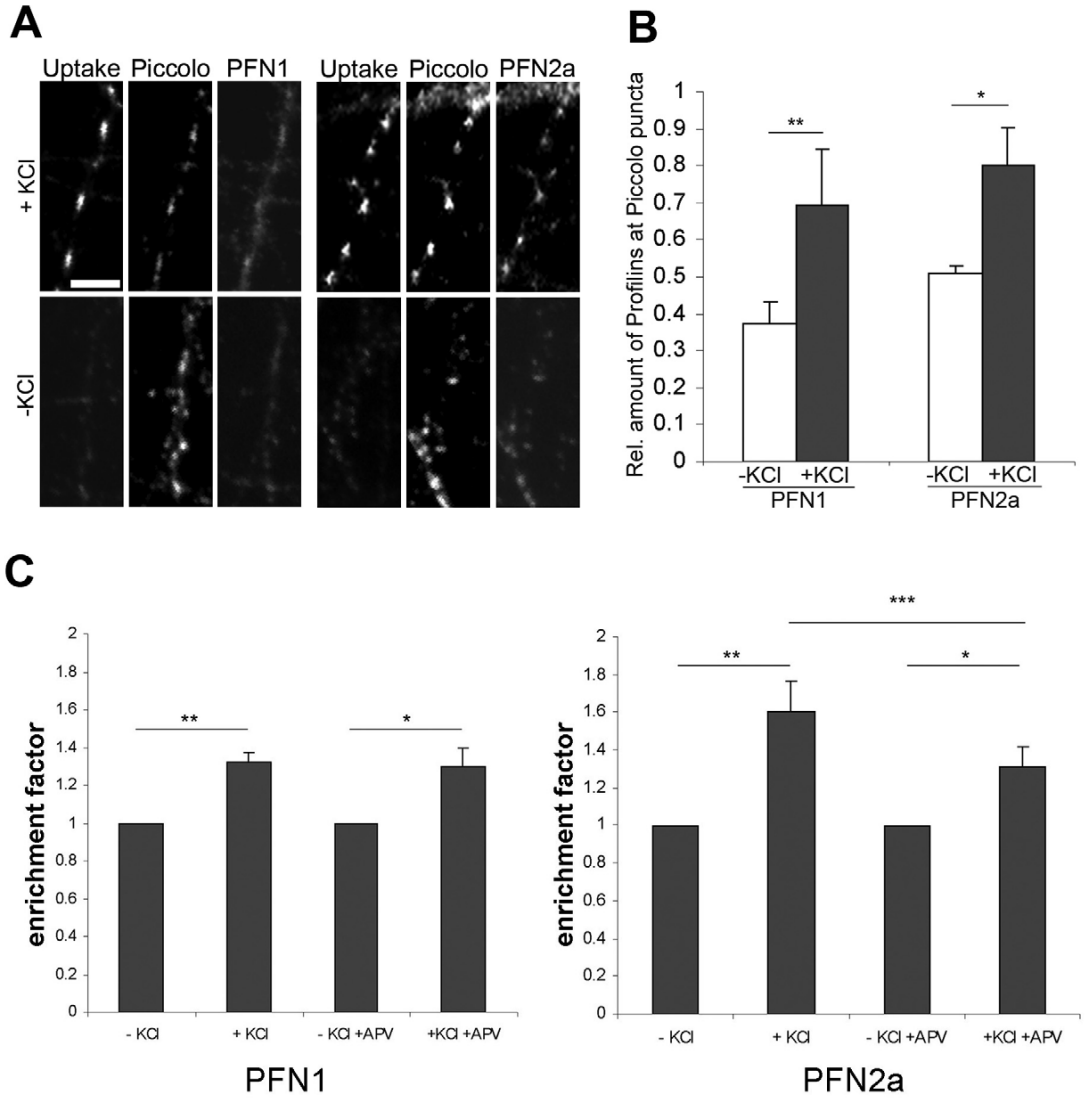
Both isoforms are enriched in activated synapses. (A): Triple immunofluorescence images of cultured rat neurons either stimulated with 70 mM KCl or kept under control conditions (-KCl), while incubated with antibodies against synaptotagmin 1. The total population of synapses was identified by staining for piccolo (centre panels), while synapses with active synaptotagmin 1 uptake were identified by staining with the corresponding antibody (uptake, left panels). Staining for PFN1 or PFN2a is shown in the right panels. Note that active synapses display a much more prominent signal for PFN1 and PFN2a than inactive ones. Bar 5 µm. (B): Quantitative analysis of activity-dependent changes in PFN1- (left) or PFN2a- (right) immunoreactivity in piccolo-positive synapses. The immunoreactivity of piccolo is independent of synaptic activity and thus serves as a reference parameter for synaptic PFN1 and PFN2a. The Y-axis reflects the relative amounts of profilin at piccolo puncta. The graph shows the mean ± SEM, n = 200–500 terminals were analysed in 2–3 independent experiments; *P<0.05, **P<0.01, statistical analysis by paired *t* test. (C): Quantitative analysis of PFN1 and PFN2a enrichment (i.e., the fluorescence signal at a synapse divided by this signal in the neurite) in dendritic spines of hippocampal neurons after KCl stimulation with and without blocking of the postsynaptic (dendritic) NMDA-receptor by the antagonist APV (100 µM). Note that inhibition of the NMDA receptor did not alter the level of PFN1 in dendritic spines, whereas PFN2a was significantly reduced. The graph shows the mean ± SEM, n = 198–296 synapses were analysed; * P<0.05, ** P<0.01, *** P<0.005, statistical analysis by paired *t* test.

To study whether the synaptic presence of profilins is subject to activity changes, we induced global synaptic activity in hippocampal rat neurons (DIV15) by high K^+^-depolarisation, and stained for piccolo and the two profilin isoforms (Figure 5). Piccolo, an active zone protein is unresponsive to activity modulation [26] and thus serves as a marker for all synaptic terminals irrespective of their activity level. To visualise selectively those synapses that are actively undergoing cycles of exo- and endocytosis high K^+^-depolarisation was performed in the presence of an antibody directed against the luminal domain of the synaptic vesicle (SV) protein synaptotagmin 1. Labelling synaptic structures by such an antibody serves as a faithful marker of synaptic activity, as it is only taken up when SVs undergo cycles of exo- and endocytosis [27], [28]. The immunofluorescence images in Figure 5A clearly show that the signal strength for both profilin isoforms markedly increases after KCl mediated activity stimulation. Quantification of fluorescence intensities of PFN1 and PFN2a immunoreactivity revealed a significant increase of PFN1 and PFN2a at synapses upon synaptic activity (Figure 5B).

Because an NMDA receptor-dependent translocation of PFN2a into dendritic spines has been described for neurons that overexpress PFN2a [17], we examined whether also endogenous profilins correspond accordingly to NMDA receptor activity. Neurons were stimulated with high K^+^ in the presence of the NMDA receptor antagonist APV. They were simultaneously immunostained for PFN1 and PFN2a, and synapses were identified by labelling with anti-synapsin. The fluorescence signal at a synapse divided by the neurite level was used to calculate the enrichment factor, which confirmed that both profilins are enriched in synapses after KCl stimulation (Figure 5C). Consistent with the data obtained for dendritic spines in neurons overexpressing PFN2a [17], blocking of NMDA receptors by APV resulted in a significant decrease of PFN2a in synaptic structures. However, this effect was not seen for PFN1 (Figure 5C). This indicates that the response of PFN1 to increased neuronal activity is not linked to NMDA receptor activation.

**Figure 6:**
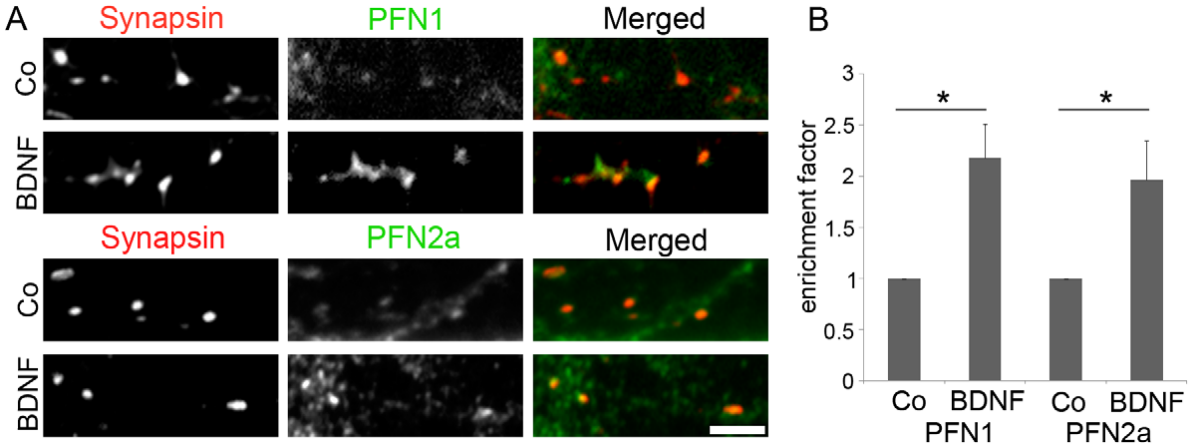
BDNF treatment results in an enrichment of both profilin isoforms in synapses of hippocampal neurons. (A): Rat hippocampal neurons at DIV9 were treated with BDNF or BSA as control (Co) for 22 hours, fixed and stained for the synaptic marker synapsin, PFN1 or PFN2a. Bar: 3 µm. (B): Quantitative analysis of the enrichment factor. 250 to 400 synapses were quantified in 3 independent experiments for each PFN isoform; mean ± SEM; paired *t* test; * P<0.05.

These data suggest that PFN1 is involved in a signalling pathway that is also triggered by KCl but different from the NMDA receptor cascade. Several receptors of neurotrophic factors are well-known modulators of neuronal morphology and activity dependent structural plasticity. For example, neuronal activation by high KCl stimulates the secretion of BDNF [29], and binding of this neurotrophin to TrkB receptors at the pre- and postsynaptic site will then trigger a large variety of signalling pathways [30]. This can lead to structural changes of synapses that are most likely mediated by the actin cytoskeleton [31], [32]. To address the question whether profilin isoforms could account for BDNF-dependent morphological alterations we activated the TrkB receptor pathway. Therefore, we stimulated hippocampal neurons with BDNF, immunostained them for either PFN1 or PFN2a and synapsin (Figure 6A), and calculated the enrichment factor for profilin at synapses. The data clearly show that BDNF stimulation caused a significant increase in synaptic PFN1 and PFN2a (Figure 6 B) and that, in contrast to the NMDA receptor activity, both isoforms respond to BDNF-induced TrkB receptor activity in the same manner.

### PFN1 and PFN2a are both constituents of neuronal nuclei

**Figure 7:**
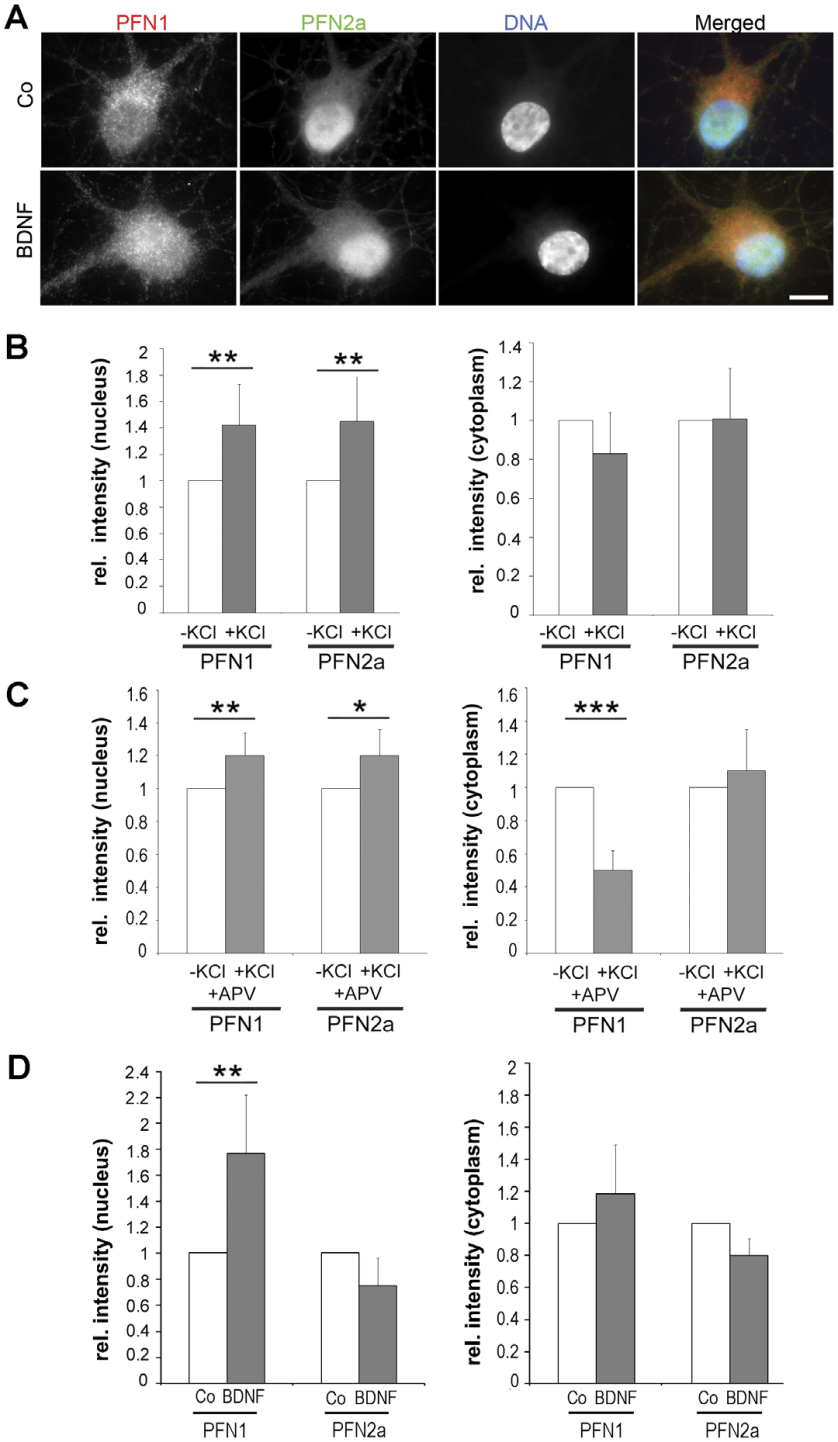
BDNF treatment results in an enrichment of PFN1 in nuclei of rat hippocampal neurons. (A): Images of non-stimulated (co) and stimulated (BDNF) hippocampal neuronal cell bodies stained for PFN1 and PFN2a and counterstained with DAPI. Bar: 10 µm. (B, C): Rat hippocampal neurons at DIV14 were treated with KCl in the presence and absence of the antagonist APV. Statistical analysis of profilin levels in the nucleus and the cytoplasm as monitored by immunofluorescence, in relation to synaptic activity. (B): fluorescence intensity of nuclear and cytoplasmic areas with and without KCl stimulation. (C): Analogous analysis with and without blocking the postsynaptic NMDA receptor by the antagonist APV. Paired *t* test; * P<0.05, ** P<0.01, *** P<0.005 (28–60 cells (B), respectively 100–129 (C) cells from 2 to 3 independent experiments were analysed). (D): Fluorescence intensity in the nuclear and the cytoplasmic compartment of neurons treated with BDNF or BSA (co) at DIV9. Paired *t* test; ** P<0.01 (30–50 cells from 3 independent experiments were analysed).

Since trafficking of profilin to the nucleus [18] and its interaction with nuclear ligands [33], [34], [35], [36] have been reported, we specifically examined the nuclear compartment of cultivated neurons for the presence of PFN1 and PFN2a. Immunofluorescence and quantitation of the fluorescence signals in the cytosol and the nucleus were performed (Figure 7). Both isoforms were present in the nucleus of non-stimulated neurons (Figure 7A). The signal for PFN1 was generally higher in the cytosol than in the nucleus, while PFN2a was frequently enriched in the nucleus (Figure 7A, control). Comparison of the respective fluorescence intensity in nuclei and cytosol of stimulated and unstimulated neurons showed that KCl stimulation significantly increased the nuclear level of PFN1 and PFN2a by 40%, while the amounts in the cytoplasm remained constant (Figure 7B). Blocking the NMDA receptor by APV (Figure 7C) resulted in a slight increase (20%) of both isoforms in the nucleus, with a concomitant decrease of the cytoplasmic level of PFN1 (50%; P<0.005), while the amount of cytoplasmic PFN2a remained almost constant (Figure 7C). Subsequently, we asked whether activation of the TrkB receptor pathway could alter the amount of profilins in the nucleus. Consistent with the effect on synaptic profilins, the addition of BDNF caused a significant rise in nuclear PFN1 by 80%, whereas no significant alterations in the cytoplasmic and nuclear level of PFN2a were seen (Figure 7A,D).

Taken together, these data indicate that BDNF stimulation led to an increase of both profilin isoforms in the synapse concomitant with a significant rise of nuclear PFN1.

## Discussion

Here we present a detailed study on the differential localisation of the profilin isoforms PFN1 and PFN2a in cultured embryonic neurons and in the adult brain of mouse and rat. In contrast to previous studies that used either overexpressed profilins [17], [18] or focussed selectively on one profilin isoform [8], [10], [14], [16], we assessed the localisation and the level of both endogenous profilin isoforms within one neuronal compartment. By means of two monoclonal mouse antibodies, we detected PFN1 and PFN2a in the same synaptic structure and in neuronal nuclei. PFN2a preferentially accumulates in discrete patterns in the majority of synapses, whereas PFN1 is only rarely enriched in synapses as compared to the neurite. This differential distribution is in agreement with a previous study that focused on endogenous PFN1 and demonstrated a high amount of this isoform in only a minority of synapses of cultured neurons and also in the adult brain [10]. These findings raise the question whether PFN1 has a specific role in a subpopulation of so far undefined neurons or might only be present in a subtype of synapses with e.g. different postsynaptic structures, e.g. different spine types.

Our ultrastructural analysis of different areas of the adult brain by immunogold labelling revealed that both PFNs are present in pre- and postsynaptic compartments, with a distinctly more intense labelling of the latter. This is consistent with a biochemical study describing a high amount of PFN2a in isolated postsynaptic density (PSD) fractions [37]. However, a lack of PFN2a enrichment in postsynaptic fractions has also been reported [8]. Conceivably, the small, highly soluble profilins might be partially lost during cell fractionation and thus, our ultrastructural analyses more faithfully reflect the *in vivo* situation than biochemical studies.

Considering the role of profilin as modulator of actin dynamics in nerve endings, the close proximity of PFN1 and PFN2a to postsynaptic densities is well in line with recent studies that demonstrate the presence of a large pool of dynamic actin in these areas [38], [39]. Furthermore, the prominent amounts of PFN2a in postsynaptic structures throughout the cortex, hippocampus and cerebellum suggest an important contribution of PFN2a to physiological functions involving the postsynapse, although the complex behavioural phenotype of *pfn*2-deficient mice has been mainly attributed to defects in presynaptic membrane trafficking [8], [16].

The presence of profilins in inhibitory synapses was observed before (see [2]) and suggests their participation in the regulation of neurotransmitter receptor organisation and mobility at the postsynaptic density, as executed by the actin cytoskeleton [1]. In addition to directly modulating actin dynamics in concert with actin nucleators such as Ena/VASP and formins, profilin can form complexes with gephyrin, a structural component of the postsynaptic zone in inhibitory synapses [11], [12]. There, gephyrin is organised in clusters. Size and geometry of these structures are regulated by the actin cytoskeleton, and determine the mobility of glycine receptors and thus the efficiency of inhibitory signal transmission [40], [41]. Our study now provides detailed information on the distribution of both isoforms in inhibitory synapses. Although recombinant PFN1 and PFN2a can bind gephyrin with comparable affinities *in vitro* [12], we found preferentially PFN2a within the gephyrin clusters of inhibitory postsynaptic structures. This finding suggests a specific role for PFN2a in inhibitory signal transmission. Considering the functions of PFN2a in dendritic spines [20] and kainate receptor dynamics [14], it is conceivable that PFN2a may also modulate other aspects in inhibitory synapses like the size and mobility of postsynaptic clusters. Notably, in spinal cords, of PFN2a knockout mice the number and density of gephyrin and glycine receptor clusters appear unaffected [8]. Clearly, further studies are required to unravel the nature of PFN2a functions in inhibitory synapses.

It is undisputed that the activity and structural plasticity of neurons is related to the organization and dynamics of actin in synapses, particularly in dendritic spines [38], [39], [42]. Being potent regulators of actin dynamics, profilins were thus assumed to respond to neuronal activity in general, and indeed, several studies have shown that the profilin levels in synaptic compartments change with neuronal activity. For example, a significant rise in the number of PFN1-positive synapses after KCl-based activation was reported [10], and GFP-PFN2a targets to dendritic spines in an activity-dependent manner [17]. In our current study we show that both, PFN1 and PFN2a levels increase in KCl-activated synapses but only PFN2a levels responded to NMDA receptor activity. Hence, PFN1 and PFN2a address different signalling pathways thus must have differential roles in the actin-dependent regulation of neuronal architecture. One example of such a differential role was already provided in our recent analysis of dissociated neurons with altered profilin levels: PFN2a deficient neurons display a significant reduction in dendritic complexity and spine numbers. Only the latter can be rescued by overexpression of PFN1 [20]. This study also revealed a differential association of PFN1 and PFN2a with p75^NTR^, a neurotrophin receptor engaged in neuronal morphology by regulating the ROCK signalling pathway [43]. In neurons displaying severe morphological changes induced by p75^NTR^ overexpression, exogenous PFN1 or PFN2a rescues either dendritic morphology or spine numbers, respectively [20]. We now report on the association between profilins and an additional pathway: The activation of BNDF dependent signalling pathways changes the levels of both isoforms in synapses. Hence, our data reveal the differential response of both isoforms to different signals, which demonstrates the importance of these isoforms for structural complexity of neurons.

Based on our data we cannot distinguish whether the elevated levels of profilin are caused by recruitment or increased synthesis. Blocking protein synthesis as a means to discriminate between these alternatives was not possible, since the incubation time required for a clear BDNF effect was 22 h, and neurons would not tolerate inhibition of protein synthesis for such a long period of time. However, a translocation of profilins induced by BDNF seems possible, regarding the fact that the BDNF ligand TrkB activates a signalling cascade, upstream of profilin. BDNF bound TrkB receptors activate phospholipase Cγ1 [44], [45] which then is capable of releasing membrane-bound profilin by PIP_2_ hydrolysation [46], [47]. And indeed a BDNF-dependent change in spine structure has been described and is dependent on TrkB activation ([32], see also [31]). In case of nuclear profilins, new synthesis seems at least in part to contribute to nuclear PFN1 after BDNF treatment, since the observed increase is not matched by a simultaneous decrease of cytoplasmic PFN1 (Figure 7D).

The presence of profilins in the nuclear compartment was described for cultured mammalian epithelial [48] and neuronal cells [18], [49] as well as for oocytes [50]. Accordingly, the nucleus harbours a number of profilin ligands including nuclear actin [51], splicing regulators [33], [35], [49] and transcription factors [34]. Here, we report that both, PFN1 and PFN2a are localized within a neuronal nucleus, and that their respective levels respond differentially to changes in neuronal activity. The functional significance of their presence in the nucleus and a possible link to synaptic transmission [18] needs to be addressed in future studies.

## Materials and Methods

### Ethics Statement

The experimental protocols were carried out in accordance with the Directive 2010/63/EU of the European Parliament and the Council of the European Union of 22 September 2010 and all procedures were approved by guidelines from the Animal Committee on Ethics in the Care and Use of Laboratory Animals of TU Braunschweig, Germany (033.42502-05-A-002/08).

### Cell culture, neuronal stimulation and transfection

C2C12 myoblasts (ATCC: #CRL-1772) were grown according to standard procedures. Primary cultures of mouse hippocampal neurons were prepared from mouse embryos E18. Embryos were decapitated and the brains were kept in ice-cold Gey's balanced salt solution supplemented with glucose. After dissection the hippocampi were dissociated by 30 min incubation with trypsin followed by mechanical separation using a Pasteur pipette. 10^5^ Cells were seeded on poly-L-lysine coated cover slips and incubated in Neurobasal medium (Invitrogen, Karlsruhe, Germany) supplemented with 2% B27 (Invitrogen) and 0.5 mM Glutamax at 37°C, 5% CO_2_ and 99% humidity. Cultures of rat hippocampal neurons were prepared from E19 rat embryos by a similar procedure [52] and cultivated for 14 days before fixation.

To study activity-dependent changes in PFN isoform localisation, three different protocols were used. (i) Dissociated rat neurons were stimulated by depolarization with KCl, and activated synapses were identified by the uptake of synaptotagmin antibodies. Neurons were stimulated with 70 mM KCl in depolarization buffer (44 mM NaCl, 2 mM CaCl_2_, 1 mM MgCl_2_, 20 mM Hepes, 30 mM glucose, pH 7.4) in the presence of synaptotagmin1 antibodies for 90 sec, followed by 10 min incubation with synaptotagmin1 antibodies in Neurobasal medium. After three washes with medium, neurons were fixed and stained for the uptake of synaptotagmin1 antibodies, for PFN1 or PFN2a, and piccolo as described below. (ii) To study NMDA receptor dependent changes in PFN isoform distribution, dissociated rat neurons were stimulated with 10 µM N-methyl-D-aspartate (NMDA, Sigma-Aldrich, Munich, Germany) in Tyrode's solution for 30 min, as described [17], [18]. The basic level of stimulation was controlled by 30 min preincubation with 100 µM (2R)-amino-5-phosphonovaleric acid (APV, Sigma-Aldrich), as described [17], [18]. (iii) Stimulation with the brain derived neurotrophic factor BDNF was achieved by exposing the cultures to BDNF (100 ng/ml, 22 h; R&D Systems, Minneapolis, MN) at DIV9 as described [53], [54]. For control treatment 1% BSA was added to the medium. C2C12 myoblasts were transfected with pEGFP-PFN2a using Fugene 6 (Roche Diagnostics, Mannheim, Germany), accordingly to manufacturer's instructions.

### Antibodies

The PFN isoform-specific mouse monoclonal antibodies used in this study were produced in our laboratory, according to standard protocols. Hybridoma culture supernatants were used for immunohistochemistry at the dilutions indicated. The monoclonal PFN1 antibody (2C5; 1∶10) was raised against a peptide sequence corresponding to aa 5–18 of mouse PFN1, which was exposed on the surface of self-assembling nanoparticles as described in [21]. The PFN2a monoclonal antibody (4H5; 1∶75) was raised against genuine PFN2a purified from bovine brain and characterized in our laboratory [5]. The monoclonal antibody against gephyrin (3B11; 1∶50) was also raised and characterised in our laboratory [55]. A monoclonal synaptophysin antibody (Sigma-Aldrich) and the following polyclonal antibodies were used as neuronal markers: Rabbit anti-synapsin and guinea pig anti-synapsin 1/2 (Synaptic Systems, Göttingen, Germany, diluted 1∶100 and 1∶500, respectively), rabbit anti-VGAT (Synaptic Systems, 1∶100), rabbit anti-piccolo (Synaptic Systems, 1∶500), and guinea pig anti-synaptotagmin 1 (Synaptic Systems, 1∶100). For indirect labelling, the following secondary antibodies were used: Cross-adsorbed goat anti-mouse IgG-Cy3 (Dianova, 1∶100), goat anti-rabbit IgG-Cy2 (Dianova, 1∶100), goat-anti guinea pig IgG-Alexa488 und -Alexa549 (Mobitec Göttingen, Germany, 1∶100) and goat anti-rabbit IgG-Cy5 (Dianova, 1∶100). To simultaneously label both profilin isoforms in the same cell, one profilin antibody was conjugated to the fluorophore by means of the anti-mouse IgG1-Alexa488 Zenon-Kit (Invitrogen), prior to using it in standard immunostaining protocols (see below).

### Immunohistochemistry, Image Acquisition and Analysis

For indirect immunofluorescence, cultures of dispersed mouse and rat hippocampal cells were fixed with 4% (w/v) formaldehyde (FA) in phosphate-buffered saline (PBS) for 20 min, permeabilised with 0.02% (v/v) Triton X-100 for 3 min, and then blocked with 1% (w/v) bovine serum albumin (Applichem, Darmstadt, Germany) in PBS. For staining of individual PFN isoforms in combination with neuronal markers, the fixed cultures were incubated with the respective primary antibodies or serum for 60 min, followed by secondary antibodies for 45 min. Staining with DAPI (Sigma-Aldrich, 1∶1000) was performed afterwards for 5 min in PBS where indicated. Images were acquired using a SPOT cooled CCD camera (Visitron Systems, Puchheim, Germany) attached to a Zeiss Axiovert 200 M microscope (Zeiss, Göttingen, Germany). Images were collected using 63× and 100× Plan-Neofluar oil objectives and digital images were analysed with Metamorph (Molecular Devices, Sunnyvale, USA). To quantify fluorescent intensities, we applied equal thresholds to all images within the respective experiment. Prior to the implementation of thresholds, background intensity was subtracted. Fluorescent intensity was measured in a defined area within the synaptic terminal and for enrichment factor calculation in the dendrite. For comparative measurements of nuclear and cytosolic profilin, the outlines of nuclei were determined by DAPI staining and cytoplasmic regions of neurite branching were not taken into account.

Simultaneous labelling with two mouse monoclonal antibodies was performed as follows. After indirect staining of the first antigen, the second antigen was labelled with fluorochromed Fab fragments bound to the Fc-portion of the respective primary antibody. These complexes, “Zenon-Fab fragments”, were prepared according to the manufacturer's protocol (Zenon Manual, Invitrogen). Briefly, 1 µg of the primary antibody was incubated with 6 µg of the Zenon-labelling solution for 5 minutes. After adsorbing unbound fluorochromed Fab-fragments by the addition of unspecific mouse IgG for 5 minutes, the now labelled primary monoclonal antibodies were added to the coverslips and incubated for 30 minutes. Unbound antibodies were removed by washing three times with PBS. Translocation of Alexa-488 conjugated Zenon Fab-fragments in the cells was prevented by additional fixation with 4% FA in PBS for 15 minutes. The specificity of this method was confirmed in control experiments showing a discrete labelling with two mouse monoclonal antibodies of a cytoplasmic and a mitochondrial protein in the same cell (data not shown). Cells were examined by confocal laser scanning microscopy (LSM 510 Meta, Zeiss, Göttingen, Germany), by using multitrack technique to monitor individual channels separately.

The PFN localisation in the mature rat brain (hippocampus, cortex and cerebellum) was examined at the ultrastructural level by immunoelectron microscopy. Adult rats (Wistar) were anaesthetized and perfused with PBS, followed by 4% (w/v) FA in PBS. The brain was dissected and cut into 100 µm sagittal sections on a Vibratome. The sections were cryoprotected in 30% (w/v) sucrose, repeatedly frozen and thawed, collected in PBS, processed free-floating, blocked with 5% FCS in PBS for 1 h and then incubated with the PFN antibodies 2C5 or 4H5 and with anti-synaptophysin. Subsequently, the sections were incubated with rabbit-anti-mouse IgG for 5 h, washed in PBS and incubated with Protein A-gold (5 nm) overnight at 4°C. After an additional wash with PBS, the sections were postfixed with 1% osmium tetroxide, dehydrated and flat-embedded in Epon 812, prior to cutting 90 nm sections on a Reichert Ultracut S (Leica) microtome. Specimens were examined in a LEO 906 electron microscope (Zeiss, Oberkochen, Germany). For quantification synapses positive for gold particles at the pre-or postsynaptic compartment and at both compartments simultaneously were included for analysis.
